# Examining the effect of training with a teaching for understanding framework on intravenous therapy administration’s knowledge, performance, and satisfaction of nursing students: a non-randomized controlled study

**DOI:** 10.1186/s12912-024-01783-6

**Published:** 2024-02-07

**Authors:** Jing Huang, Xiaoyan Liu, Jing Xu, Li Ren, Lihui Liu, Ting Jiang, Menglu Huang, Zhoupeng Wu

**Affiliations:** 1 Department of Vascular, Department of General Surgery, West China Hospital, Sichuan University, Chengdu, 610041 Sichuan China; 2 Department of Orthopedic Surgery, West China Hospital, Sichuan University, Chengdu, 610041 Sichuan China; 3 School of Environmental Science and Engineering, Shanghai Jiao Tong University, Shanghai, 201100 China; 4 West China of Nursing, Sichuan University, Chengdu, 610041 Sichuan China

**Keywords:** Teaching for understanding framework, Infusions, Intravenous, Students, Nurses, Teaching

## Abstract

**Background:**

Nursing students require improvement in their intravenous infusion therapy management skills, yet traditional training models possess deficiencies. The Teaching for Understanding (TfU) Framework can enhance the teaching-learning process and support quality education. Therefore, utilizing TfU framework for training may promote the performance of nurses.

**Methods:**

Utilizing a non-synchronized design, 102 nurses were recruited using a convenience sampling method. Fifty-one student nurses from August 2019 to January 2021 were designated as the control group, and 51 student nurses from February 2021 to July 2022 were included as the intervention group. The control group received traditional teaching methods, while the intervention group was trained based on TfU framework. The impact was gauged through medical education environment perception, theory and practice assessments, and learning satisfaction surveys.

**Results:**

After the training, there was no significant difference between the control group and the intervention group in the theory assessment. However, the practice assessment scores of the intervention group were significantly higher than those of the control group. Compared with the control group, the learning satisfaction scores of the trained nurses in the intervention group were significantly higher, exhibiting significant differences, particularly in communication ability, teamwork cooperation, summing up capability, and interest in learning improvement. Furthermore, the scores of the learning perceptions, atmosphere, social self-perceptions, and total scores of the intervention group were significantly higher.

**Conclusion:**

Training using TfU framework can heighten students’ understanding and command over knowledge and skills, fuel their learning fervor, and enhance their communication and collaboration abilities. TfU framework should be disseminated in medical education to improve the quality of education.

## Background

Intravenous (IV) infusion is a common nursing operation employed in various healthcare settings for managing fluid, electrolyte, and acid-base imbalances [1]. Studies report that more than 70% of inpatients are treated via peripheral intravenous catheters [2]. In China, the amount of IV infusion was as high as 13.692 billion bottles (bags) with the infusion rate of inpatients over 94% in 2014 [3]. Moreover, complications including tube blockage, tube disconnection, fluid extravasation, phlebitis, and infection, are also costly to the healthcare system [3, 4]. As most of the nurses will be involved in infusion care [5], improperly trained nurses have the potential to adversely affect patients [6–8]. Nonetheless, complications were preventable when nursing students gained adequate training during their education [9]. Therefore, there is an urgent need for educational measures aimed at lowering the number of complications connected to infusion therapy.

Although infusion is frequently performed, nursing students receive little training or opportunity to practice the related skills [8, 10]. With the increase in the sense of authority in directing their medical care, patients may not want to be subjected to initial practical exercises by student nurses. As a result, nursing practitioners are not fully proficient in their skills as practical opportunities for clinical training are increasingly difficult to obtain.

Classroom sessions including lectures, case analysis, and training under the supervision of a teacher were provided in the teaching pedagogies conventionally. But the planning and implementation of intravenous infusions require clinical judgement, interpretation by the nurse, and modification according to the patient’s response [11]. Therefore, the flexibility and variability in clinical work can result in a gap between theory and practice. New instructional methods and modalities should be used to help nursing students to develop these skills.

Selecting a framework based on the conditions is one of the most important steps in educational planning [12]. The Teaching for Understanding (TfU) Framework advocates providing rich material learning environments, offering ample opportunities for interaction and engagement, and leading to further development, which was used to reach quality in education [13]. One of the TfU frameworks is “Putting Understanding Up Front” [14], which is a four-part framework that highlights four key concepts: generative topics, understanding goals, understanding performances, and ongoing assessment. The TfU framework emphasizes understanding and personalized assessment, promoting deep learning for students, but may require more time and resources and pose challenges to teacher skills. A review aimed to analyze the relationship between the European Competency and TfU framework conceived that the TfU framework proposes a structure with enough versatility to cover the needs of teachers and students regarding the classroom processes, and can improve the teaching-learning process [13]. In addition, a study conducted in gastrointestinal surgical department showed the use of the TfU framework can increase the core ability, knowledge, and performance of nurses, and ultimately extends the quality of treatment and patient safety [15].

Although nursing students get excellent theoretical instruction, the prevalence of issues related to patient safety and numerous students may make it challenging for them to develop these practical skills during clinical practice. It is time to reconsider conventional teaching methods and implement deliberate changes to the teaching-learning process. There are no previous studies about intravenous training with TfU framework in China.

Therefore, this study aimed to investigate in the effect of training with the four-part framework on IV therapy administration knowledge, performance, and learning satisfaction of nursing students.

## Methods

### Design and setting

The study focuses on a historical control cohort of student nurses in the vascular surgery department of a tertiary university hospital in China. The intervention group received intravenous therapy training based on TfU framework from August 2021 to July 2022. The control group was trained in routine from August 2019 to January 2020. Both groups were trained according to the syllabus made by our tertiary university hospital’s nursing team.

### Participants

Nurses were recruited who trained in our hospital work in groups of 5 for 3 weeks at a time. The study targeted of 102 student nurses (20 batches) trained in our hospitals. Fifty-one student nurses (10 batches) from August 2019 to January 2021 were designated as the control group, and 51 student nurses (10 batches) from February 2021 to July 2022 were included as the intervention group.

### Inclusion criteria

All participants should meet the following criteria: (1) students with no abnormal physical or mental symptoms now; (2) full-time undergraduates; (3) age ≥ 18 years old; (4) voluntary participation in this study signed informed consent.

### Exclusion criteria

Participants who did not want to continue studying, did not show up for the program, and did not finish the pre- or post-test were excluded.

### Sample size and sampling method

The sample size was calculated based on the trial conducted by Uzelli Yilmaz & Sari [16]; 88 participants were required for each group, with α = 0.05 and β = 0.10. Considering a dropout rate of 15%, the sample size needed was 102. A convenience sampling method was used to select participants. Students who were enrolled during August 2019 to January 2021 and who agreed to participate were recruited to the control group, while those enrolled during February 2021 to July 2022 were recruited to the intervention group with their consent.

### Measures and data collection

#### Knowledge test for the IV therapy

The theoretical examination was designed by peer experts and included single-choice, multiple-choice, noun explanation, and short answer questions. Topics covered in the test include venous anatomy, venous access and infusion tool selection, common intravenous infusion drugs, common complications of intravenous infusion, quality evaluation of intravenous treatment, and occupational protection. The highest score possible was 100, and the lowest was 0.

#### Performance evaluation for IV infusion with IV catheter insertion

The performance evaluation form consisting of 25 steps was designed by the researchers, and used by teachers. The highest score was 100, and the lowest score was 0. This self-made test demonstrated good reliability and validity in our study.

### Educational environment assessment

The Dundee Ready Education Environment Measure [17] was used to evaluate the students’ educational environment and learning effects, which includes five dimensions: students’ perception of learning (SPL) (12 items), students’ perceptions of teachers (SPT) (11 items), students’ academic self-perceptions (SASP) (8 items), students’ perceptions of atmosphere (SPA) (12 items), and students’ social self-perceptions (SSSP) (7 items). Items were scored by Likert Grade 5 (0 for total disagree and 4 for total agree), among which items 4, 8, 9, 17, 25, 35, 39, 48, and 50 were reverse scores. There were 50 items on the scale, with a perfect score of 200. A higher score represented a better educational environment students perceived. The Cronbach’s alpha value of the questionnaire was 0.90, indicating a high reliability of the questionnaire.

### Evaluation of learning satisfaction

The learning satisfaction evaluation questionnaire [18] includes 10 items to rate the level of learning satisfaction with students’ attitudes toward training. To assess the training’s effects on the improvement of theoretical knowledge, communication ability, practical ability, logical thinking ability, writing ability, team cooperation ability, literature review ability, summarization ability, learning interest, and learning efficiency, Likert 10 grade scoring method was adopted. The highest score of each item was 10 points consisting of a total score of 100 points. A higher score indicated greater satisfaction among students. The Cronbach’s alpha value of the questionnaire was 0.89, indicating a high reliability.

### Data collection procedure

After completing the training, all students were given a questionnaire survey to evaluate the Dundee Ready Education Environment and their learning satisfaction. Knowledge and performance tests were completed during the final week of the students’ internship.

### Intervention

**Fig. 1:**
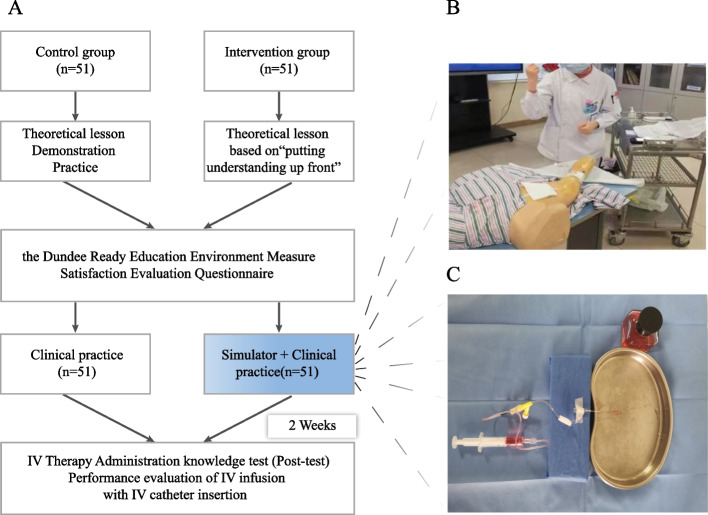
The flow chart of the training process (**a**). Students are accompanied by a teacher during the practical performance (**b**). The tube flushing and sealing simulator (**c**)

A quasi-experimental design was employed in this study. (Fig. 1a).

The intervention was conducted for 1 week. The curriculum (detailed in Table 1) was designed based on the “Infusion Therapy Standards of Practice, 8th Edition.” It covers topics such as venous anatomy, selection of venous access and infusion tools, common intravenous infusion drugs, potential complications of intravenous infusion, quality evaluation of intravenous treatment, occupational protection, interpretation of the latest “Guidelines and Detailed Rules for Nursing Practice of Infusion Therapy,” the national health industry standard “Technical Operation Specifications for Nursing of Intravenous Therapy,” the concept of active intravenous therapy, and safety management.

**Table 1:** Intravenous Therapy Training Week Plan

Time	Theoretical training	Operational training
Monday	Knowledge of venous anatomy selection of venous access, performance and characteristics of infusion tools	Intravenous infusion with indwelling needle
Tuesday	Common complications of intravenous infusion drugs	Evaluation of intravenous infusion access (evaluation object and method)
Wednesday	Evaluation and maintenance of pipeline quality evaluation of intravenous treatment, occupational protection	Maintenance of intravenous infusion access (liquid selection and technique of flushing and sealing tube)
Thursday	Interpretation of related guidelines and specifications for intravenous infusion	Maintenance of intravenous infusion access (free exercise)
Friday	Case analysis	

**Fig. 2:**
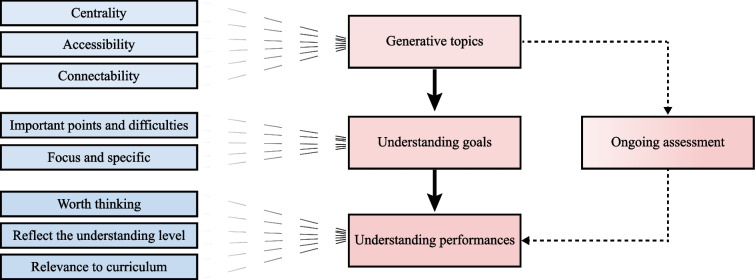
The framework of Teaching for Understanding

The control group received five sessions of IV therapy education, following the existing curriculum, through conventional methods including classroom teaching, panel discussion, and demonstrations. The experimental group received five sessions of the revised program, which was designed based on TfU framework proposed by David Perkins and Tina Blythe’s “putting understanding up front” framework (Fig. 2).

Take the curriculum of “vascular access device management” in our training as an example.

Generative topics: A generative topic consists of three features: centrality to the curriculum, accessibility to students, and connectability to various topics inside and outside the curriculum. “vascular access device management” involves flushing, sealing, locking, and removal of the vascular access device. Considering that students have learned the fundamentals of IV therapy such as the venous anatomy, selection, and placement of the venous access device but lack experience in device management, we developed a tentative generative topic: maintain the patency of the venous access device. This topic revolves evaluation of device and intravenous medication, sealing, and flushing of tubes requiring both skills and knowledge in nursing, pharmacy, and clinical medicine.

Understanding goals: Giving priority to the learning condition, starting from the characteristics of knowledge related to IV, theory and practice must be considered. The following goals are presented: What are the causes of catheter blockage? What are the possible complications of intravenous therapy?

Understanding performances: Sharing videos of establishing venous access and prompting students to consider a series of questions about potential complications during venous access construction, as well as important links that require attention, the most serious consequences that may arise, and so on. After classroom instruction, students are prompted to re-evaluate their answers to these questions. After receiving practical demonstrations, students work in groups using a tube flushing and sealing simulator. Finally, students perform tube flushing and sealing under the guidance of a teacher (Fig. 1b).

### Creation of the tube flushing and sealing simulator

The simulator with an approximate assembly time of 5 minutes includes a glass bottle filled with red ink and a plastic hose. Put one end of the hose into the glass bottle. Then connect one end with a peripheral venous catheterization device (such as an indwelling needle, PICC pipeline, etc.), and a needle-free infusion joint (Fig. 1c).

Ongoing assessment: The assessment went along with training. The review of students’ performance during quizzing in the classroom was carried out by other students and teachers. This started with everyone complementing each other in the class and ended with the teachers’ summary. During the operation on patients, they were group members who first gave advice and opinions, and all teachers had to do was encourage them and provide further explanations.

The two groups were taught by the same teachers who served as a facilitator to guide them to learn and to provide immediate assistance when students encountered problems during the learning process.

### Ethical considerations

This study was approved by the Biomedical Ethics and Committee of West China Hospital, Sichuan University (reference number 724), informed consent was obtained from participants.

### Data analysis

The data underwent a two-sided test at a significance level of 0.05 using SPSS 20.0. The data were expressed as means and standard deviations. After verifying the normality of the data and homogeneity between groups, an independent samples t-test was used to compare the differences in the effectiveness of the intervention program between the experimental and control groups.

## Results

### Characteristics of participants

All the participants were females. There was no significant difference in age between the 2 groups.

### Knowledge test and performance evaluation of the participants after the training

**Fig. 3:**
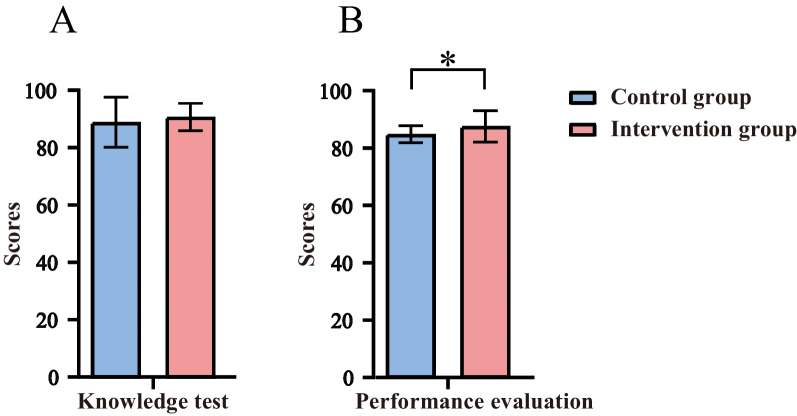
Comparison of the control group and intervention group on the knowledge test (**a**) and performance evaluation (**b**). (*The difference between the observation group and the control group was statistically significant, *p*<0.05)

The results showed that there was no significant difference in knowledge test scores between the two groups (Fig. 3a). However, the intervention group had significantly better performance evaluation scores than the control group (Fig. 3b). These results suggest that using the TfU framework for radiation therapy teaching can improve students’ practical skills and skill acquisition.

### Mean scores of educational environment assessment after the training

**Fig. 4:**
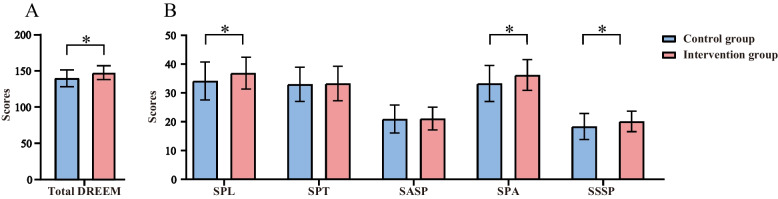
Comparison of the control group and intervention group on the overall educational environment assessment (**a**). Comparison of the control group and intervention group on students’ perception of learning (SPL), students’ perceptions of teachers (SPT), students’ academic self-perceptions (SASP), students’ perceptions of atmosphere (SPA), and students’ social self-perceptions (SSSP) (**b**). (*The difference between the observation group and the control group was statistically significant, *p*<0.05)

The intervention group had significantly higher scores than the control group on SPL, SPA, SSSP, and total DREEM (Fig. 4). This indicates that using the TfU framework for radiation therapy teaching can improve students’ perceived educational environment.

### Mean scores of learning satisfaction survey after the training

**Fig. 5:**
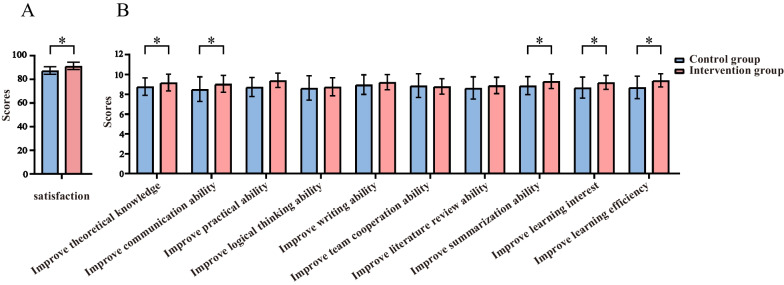
Comparison of the control group and intervention group on the overall learning satisfaction survey (**a**). Comparison of control group and intervention group on 10 dimensions of learning satisfaction survey (**b**). (*The difference between the observation group and the control group was statistically significant, *p*<0.05)

The results of the learning satisfaction survey showed that the intervention group had significantly higher scores than the control group in improving communication ability, practical ability, summing-up ability, learning efficiency, and learning interest, with statistically significant differences (Fig. 5). The overall learning satisfaction of the intervention group was better than that of the control group. In the 10-point scale questioning about whether the simulator is a useful tool for trainee education, all learners in the intervention group scored the simulator as high (between 8 and 10) in all categories. This suggests that using the TfU framework for radiation therapy teaching can increase students’ interest and satisfaction in learning.

## Discussion

Vascular access is crucial to the effective administration of medications and fluids for resuscitation. Providing health workers with training to use reliable methods was suggested before [19], and improving IV skill in trained nurses has significant effects on patient safety [20]. In the traditional teaching model, teachers demonstrate the workflow and emphasize the key points of steps through oral presentation, which makes it difficult to effectively teach the students. TfU is an approach to think about teaching and how to work with our students [13]. It provides teachers with a language and strategy for enhancing their effects to teach for greater understanding, and makes it much easier for teachers to organize and discuss their approach to a particular topic or an entire course.

In this study, the students trained with TfU framework have significantly better performance than those without (*P* < 0.05). A study investigated the effect of TfU framework on the development of nursing students’ knowledge and practice skills, and the results revealed that the knowledge and skills scores of the students trained with TfU framework were significantly higher than those trained with regular methods [15]. This study suggests that TfU framework is successful in transferring the skills acquired in the training to clinical practice. Err permission, the chance to practice, and feedback in time may prevent the repetition of mistakes on real patients [21]. The inclusion of simulators is effective way to develop students’ self-confidence, increase their self-efficiency levels [22]. Teaching aids such as a simulator for tube sealing and flushing could strengthen the students’ understanding of the operations of flushing and sealing tubes, which helped improve their operation skills. Skill-specific simulation training and other interventions may help medical students in obstacles to develop proficiency in the clinical setting and improve their skill development [23].

The mean scores of SPL, SPA, SSSP, and total DREEM were significantly higher for those trained with TfU framework. Medical education environment is the sum of all subjective and objective factors related to medical teaching [24], which can directly affect learning effects [25, 26]. TfU framework cultivates a safe learning environment, which enhances teamwork and optimizes learning [27]. The intervention group experienced a learning environment where they could communicate with standardized patients (SPs) and had fidelity with the simulator. Simulation, as one of the understanding performances, provides a low-stakes environment for skills education without exposing patients to risks associated with traditional clinical procedural training [28]. The introduction of new pedagogical practices (SPs, simulation) can improve students’ experience as practice on patients may increase the fear and restriction of students and lead to dissatisfaction among patients. By creating an operation scene similar to clinical practice, students can experience a three-dimensional environment close to clinical practice in advance. Teachers can interrupt the practice at any time, revise students constantly, and give assistance properly. Therefore, students can find their own problems in time.

The results showed that the learning satisfaction scores of students trained using TfU framework are statistically higher. Similarly, other studies have concluded that TfU framework increased the learning satisfaction scores of nursing students [15, 18, 29]. It’s probably because students from the intervention group have better training experience and closer relationships with the teachers. Study indicates that new nurses choose teachers who are open to their questions and assist them to develop proper clinical judgement and practice knowledge [30]. The relationship between students and teachers in training using TfU framework can be described as a two-way partnership between people, based on continuing support, aiming at tackling the needs, issues, and blockages identified by the students. Teachers using TfU framework to conduct the instructional design can draw upon the skills of coaching, questioning, and counselling to lead students, build up their capacity, help them discover their own wisdom, and encourage the students to achieve their goals.

The Teaching for Understanding (TfU) framework offers a valuable teaching approach that emphasizes student understanding and active learning, supported by personalized assessment and feedback. However, its implementation may demand additional time, resources, and teacher expertise. Advantages include its emphasis on deep understanding, promotion of diverse learning environments, and personalized assessment. On the other hand, challenges include increased time and resource requirements, the need for teacher expertise and skills, and operational challenges related to adaptability and flexibility.

We recommend TfU framework to all teachers. Simultaneously, there are some important things to notice about ongoing assessment in the framework. First, the assessment should not take place only at the end of the student’s placement but should be an ongoing practice akin to the nurse process approach of assessing, planning, implement, and evaluation. Assessment can be done on a one-to-one basis, or with the team, aiming to identify gaps in learning. Second, give feedback about the points the nursing students closely focus on during their work [31]. The timing of feedback is important. To encourage timely reflection, offer feedback as soon as possible after evaluating the student’s performance. Concentrate on the student’s strengths first when offering feedback, and discuss with the student how their strengths can be perfectly applied in both the present and future and how to avoid poor performance. Finally, end the feedback session with the most positive aspects of the student’s performance. In other words, criticism is sandwiched between positive and supportive news. Offer specific feedback (on strengths or weaknesses) which is descriptive rather than evaluative, and objective rather than subjective.

### Limitations

The primary limitation of this study is the absence of randomized control trials. This was due to data being collected from a single institution, with control group data collected prior to the experimental group. The sample size was not sufficiently large for conducting statistical analysis to compare mean results across different demographic data, limiting generalizability. A follow-up study using a randomized controlled trial was recommended, in which the number of research institutes and participants is expanded to generalize our results. Moreover, this study only examined the short-term effect of the framework. We look forward to further research on evaluating how the educational effect is maintained or how knowledge and skills are applied to actual nursing practice.

## Conclusion

Using TfU framework, we have successfully trained students to enhance the learning effect and improve the educational environment. The students’ learning satisfaction level has been demonstrated to be high. This indicates that the nursing education approach with TfU framework represents an effective educational strategy that increases students’ learning satisfaction and fosters students’ skill development. TfU framework calls for teachers who possess communication skills, problem-solving abilities, the ability to identify priorities, the utilization of decision-making strategies, and the capacity to provide learning opportunities for both students and teachers. Teachers should employ this framework to promote positive learning and set higher demands on their professional expertise in order to prepare for the revolution of teaching models and methods.

## Data Availability

All data generated or analyzed during this study are included in this published article.
